# Comparative effectiveness of elemental formula in the early enteral nutrition management of acute pancreatitis: a retrospective cohort study

**DOI:** 10.1186/s13613-018-0414-6

**Published:** 2018-06-05

**Authors:** Akira Endo, Atsushi Shiraishi, Kiyohide Fushimi, Kiyoshi Murata, Yasuhiro Otomo

**Affiliations:** 1 Trauma and Acute Critical Care Medical Center, Tokyo Medical and Dental University Hospital of Medicine, 1-5-45 Yushima, Bunkyo-ku, Tokyo, 113-8510 Japan; 2 Emergency and Trauma Center, Kameda Medical Center, 929 Higashicho, Kamogawa, Chiba Japan; 3 Department of Health Policy and Informatics, Tokyo Medical and Dental University Graduate School of Medicine, 1-5-45 Yushima, Bunkyo-ku, Tokyo, Japan; 4 The Shock Trauma and Emergency Medical Center, Matsudo City Hospital, 4005 Kamihongo, Matsudo, Chiba Japan

**Keywords:** Acute pancreatitis, Enteral nutrition, Elemental diet, Polymeric formula, Mortality

## Abstract

**Background:**

Although enteral nutrition has become one of the standard therapies for patients with acute pancreatitis, the optimal formulae for enteral nutrition have been under debate. Elemental formula is assumed to be suitable in the treatment of patients with acute pancreatitis because it has less stimulating effects for exocrine secretions of the pancreas, simultaneously maintaining gut immunity; however, clinical studies corroborating this assumption have been scarce.

**Methods:**

We conducted a retrospective cohort study using a Japanese national administrative database between 2010 and 2015. Patients with acute pancreatitis who received enteral feeding within 3 days of admission were identified and divided into two groups according to whether elemental formula was administered. We assessed the impact of elemental formula for the outcomes (primary, in-hospital mortality; secondary, development of sepsis, hospital-free days at 90 days, and total health-care costs) using a multivariate mixed-effect regression analysis and propensity score matching analysis adjusted by a well-validated case-mix adjustment model. Analysis for the subpopulation of patients with severe acute pancreatitis was also performed.

**Results:**

Of 243,312 patients with acute pancreatitis, 948 patients were identified and classified into the elemental formula group (*N* = 382) and the control group (*N* = 566). No significant differences were observed for in-hospital mortality [10.2% in the elemental formula group vs. 11.0% in the control group; adjusted adds ratio (95% confidence interval; CI) = 0.94 (0.53–1.67)], sepsis development [5.0 vs. 7.1%; adjusted adds ratio (95% CI) = 0.66 (0.34–1.28)], mean hospital-free days [54 days vs. 51 days; adjusted difference (95% CI) = 2 days (− 2 to 5)], and mean total health-care costs [$29,360 vs. $34,214; adjusted difference (95% CI) = − $4250 (− 8643 to 141)]. Similar results were also observed in patients with severe acute pancreatitis.

**Conclusions:**

The results of our retrospective cohort study using a large-scale national database did not demonstrate the benefit of elemental formula compared to semi-elemental and polymeric formulae in patients with acute pancreatitis. Further assessment of alternative nutritional strategy is expected.

**Electronic supplementary material:**

The online version of this article (10.1186/s13613-018-0414-6) contains supplementary material, which is available to authorized users.

## Background

Acute pancreatitis is a life-threatening inflammatory disease characterized by autodigestion and destruction of the pancreas due to self-producing proteases. The standard treatment for acute pancreatitis mainly comprises supportive therapy, such as adequate fluid resuscitation and respiratory care, because evidence of disease-specific therapy has been limited [1–3].

Adequate nutritional strategy has been one of the key factors during systematic support in patients with acute pancreatitis [4, 5]. The concept of “pancreatic rest” had been widely believed to be the standard nutritional strategy in the management of acute pancreatitis; therefore, total parenteral nutrition had been widely used up to the 1990s [6, 7]. However, several randomized controlled trials (RCTs) conducted in the late 1990s demonstrated the consistent superiority of enteral nutrition over parenteral nutrition [8–11]. Among the enteral nutrition formulae, elemental formula has been believed to elicit theoretical advantages owing to a lower degree of exocrine pancreatic stimulation and because it is fat-free. A previous RCT [12] comparing the efficacy of a semi-elemental formula with a polymeric formula was underpowered (*N* = 30) and failed to demonstrate the superiority of a semi-elemental formula. Because few RCTs or large-scale cohort studies have been reported in this theme, Petrov et al. [13] performed an indirect adjusted meta-analysis, in which parenteral nutrition groups were used as the reference and reported that significant difference regarding survival benefit and adverse events were not observed between the groups of (semi)elemental formulae and polymeric formula. However, to our knowledge, a large RCT or cohort study that directly compared the efficacy of elemental formula has not been reported in the treatment of acute pancreatitis. In this study, we aimed to assess the clinical benefit of elemental formula compared to the other formulae (semi-elemental and polymeric formulae) in the initiation of enteral nutrition management in acute pancreatitis, using a large-scale national administrative database.

## Methods

### Study design and data sources

We conducted a retrospective cohort study to evaluate the efficacy of elemental formula in patients with acute pancreatitis, using the Japanese Diagnosis Procedure Combination (DPC) database. The database is a case-mix classification system that is linked to the reimbursement system for inpatient cases in Japanese hospitals and contains administrative claims for every drug administered and every procedure and care performed at more than 1500 hospitals. Each patient’s primary diagnosis, comorbidities at admission, and post-admission complications are independently recorded using the relevant codes from the International Classification of Diseases, 10th revision (ICD-10). In addition, the DPC database includes baseline characteristics of patients and information regarding the treating hospital. Furthermore, information regarding the severity of acute pancreatitis was recorded using the Japanese severity scoring system for acute pancreatitis of the Ministry of Health, Labour, and Welfare of Japan [14], as this comprises the prognostic factor scores and computed tomography (CT) severity scores based on contrast-enhanced CT (Additional file 1). Further details regarding the DPC database have been described elsewhere [15].

This study was conducted in accordance with the principles of the 1964 Declaration of Helsinki and its later amendments. The institutional review board of the Tokyo Medical and Dental University approved this study (#788). Informed consent from each patient was waived because of the retrospective design of the study and the use of anonymized patient and hospital data.

### Study population

We included patients who were admitted to the hospital because of acute pancreatitis between April 1, 2010, and March 31, 2015, and patients who received nasogastric feeding or nasojejunal feeding within 3 days of admission. We excluded patients younger than 16 years and those who were pregnant. Patients who were discharged within 3 days of admission were also excluded considering the issue of immortal-time bias. In addition, we excluded patients who had missing values in any variables used in the analyses (i.e., complete case analyses).

### Data collection

We collected the following information from the DPC database: age; sex; ICD-10 codes for four primary diagnoses, four concurrent diagnoses at admission, and four post-admission complications; the aforementioned prognostic factor score and CT severity score; unique hospital identifier; annual number of patients with acute pancreatitis per hospital; presence or absence of specific reimbursement claims for enteral feeding; presence or absence of specific reimbursement claims for use of elemental formula; status at hospital discharge (i.e., survived or deceased); total health-care costs per admission; and length of hospital stay. Furthermore, we collected information on whether the following interventions were performed within 3 days of admission: administration of vasopressors (dopamine, norepinephrine, epinephrine, or vasopressin), mechanical ventilation, renal replacement therapy, and transfusion. Patient comorbidities were assessed using the Charlson comorbidity index [16] based on a previously reported method for extracting the ICD-10 codes [17].

### Definitions and outcomes

Patients who were administered elemental formula were identified by the presence of specific reimbursement claims of elemental formula (Elental^®^, Ajinomoto Pharmaceutical Ltd., Tokyo, Japan; and Hepan ED^®^, EA pharma Ltd., Tokyo, Japan; those are all the elemental formula products approved in Japan). The control group was defined as patients who were administered other types of enteral nutritional formulae within 3 days of admission. The primary outcome was in-hospital mortality. Secondary outcomes were development of sepsis after admission, hospital-free days, and total health-care costs per admission. The ICD-10 codes used to identify sepsis are presented in Additional file 2. Hospital-free days were defined as days survived and days free from hospitalization within 90 days from initial hospital admission. A recent clinical trial group consensus recommended that hospital-free days should represent composite measures compared to length of hospital stay, which could be highly influenced by mortality [18]. Total health-care cost was defined as all aggregated payments (except for boarding costs) to the hospital per discharge, and these payments were estimated using the reference prices in the Japanese fee schedule, which lists reimbursement rates for surgical, pharmacological, laboratory, and other inpatient services. The cost data were provided after converting the cost in yen to US dollars (100 yen = $1 USD).

### Statistical analysis

We developed a risk adjustment model for in-hospital mortality using the variables of age, sex, Charlson comorbidity index, prognostic factor score, CT severity score, mechanical ventilation within 3 days of admission, renal replacement therapy within 3 days of admission, transfusion within 3 days of admission, and vasopressor use within 3 days of admission by applying a logistic regression model that included a random sample of 80% of the entire study cohort. The covariables used were selected based on clinical experience and previous studies [19, 20]. Issues with variable multicollinearity were assessed using a variance inflation factor (VIF), and the tolerance value was set at < 2. We validated the model for the remaining 20% of the cohort using the area under the receiver operating curve (AUROC) and a Hosmer–Lemeshow goodness-of-fit test.

We then compared the outcomes between the elemental formula group and the control group using a mixed-effects logistic regression model [21] for binary outcomes and a linear-mixed regression model for continuous outcomes as the primary analysis, adjusted by the case-mix classification model established, with the random effects of hospital-level clustering. In the linear mixed-effects model, the case-mix classification model was inverse-logit-transformed to satisfy the homoscedasticity requirement for linear regression.

We further compared the outcomes in the elemental formula group and the control group using a propensity score matching analysis [22] as the secondary analysis. The propensity score for predicting administration of elemental formula was calculated through a logistic regression analysis using the variables used for the establishment of the aforementioned prognosis model and the annual number of acute pancreatitis cases per hospital as a variable to account for the differences in treatment quality at each hospital. Propensity score matching extracted 1:1 matched pairs from the elemental formula group and the control group. A match balance between the two groups was assessed using the absolute standardized mean difference (ASMD) of all variables; values < 0.1 were regarded as acceptable. To achieve balanced matching, the caliper width for matching was set as the standard deviation (SD) of the logit-transformed propensity score multiplied by 0.3. Intergroup comparison of the outcomes with propensity score-matched subjects was performed using a Chi-square test.

In addition, we performed analyses using the aforementioned two models only in patients with severe acute pancreatitis (SAP) diagnosed by the severity diagnosis criteria in Japan, to assess the efficacy of elemental formula in patients with SAP.

All statistical analyses were performed using R software (version 3.4.1; R Foundation for Statistical Computing, Vienna, Austria). The level of significance was defined as *p* < 0.05.

## Results

### Study population

**Fig. 1:**
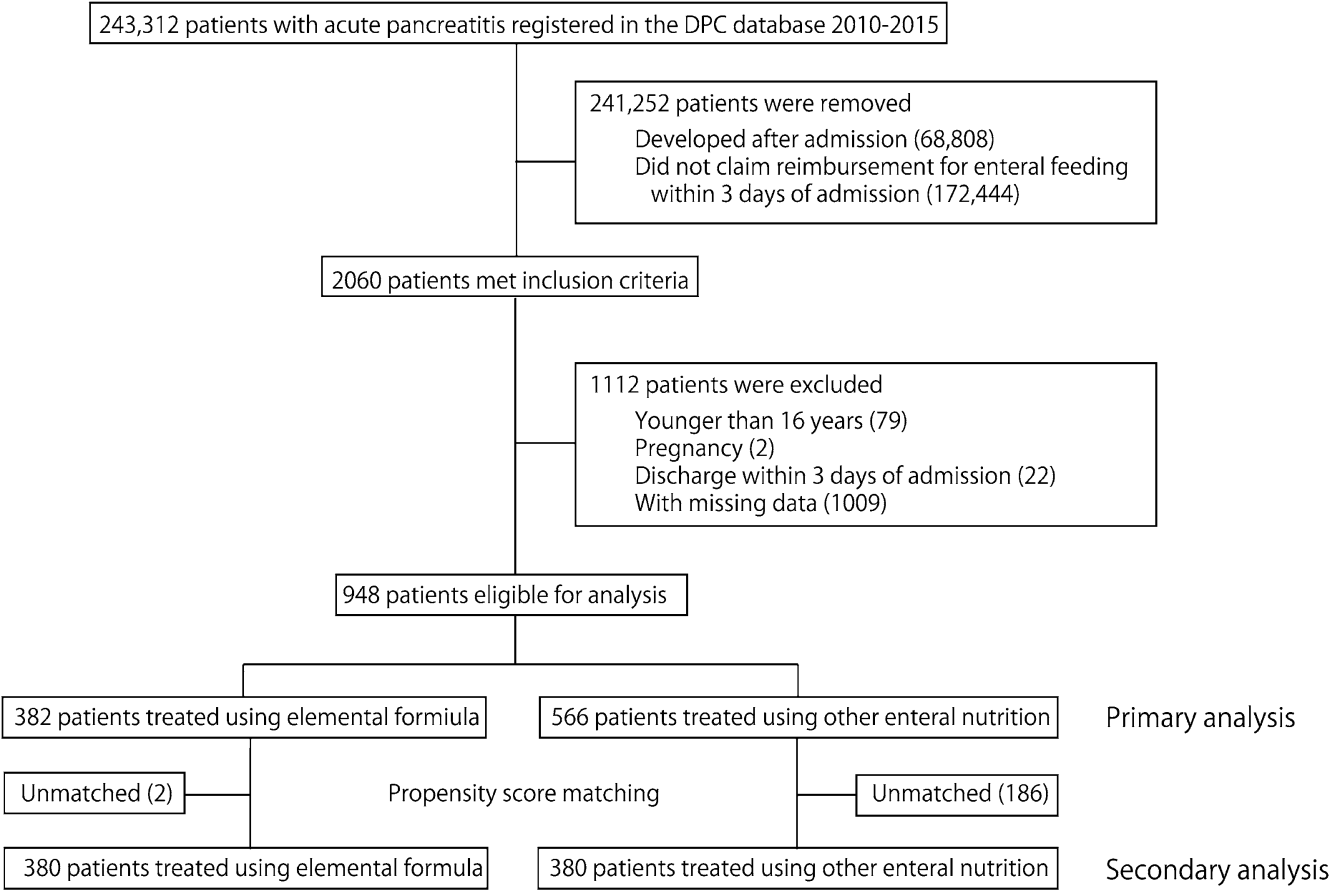
Flow diagram of patient selection. *DPC* Diagnosis Procedure Combination

The flow diagram of the patient selection process is presented in Fig. 1. During the study period, a total of 243,312 patients with acute pancreatitis were hospitalized at the DPC participating hospitals. Of these, 948 patients were identified according to the inclusion and exclusion criteria. Among these patients, 382 (approximately 40.3%) were administered elemental formula. Patients’ characteristics according to whether elemental formula was administered are presented in Table 1. The in-hospital mortality rate was 10.2% (39/382) in the elemental formula group and 11.0% (62/566) in the control group. Prescription doses of elemental formula from the day of admission to day 14 days are shown in Additional file 3. Median duration of enteral feeding (25th–75th percentiles) was 9 days (5, 17) in the elemental formula group and 10 days (5, 18) in the control group, respectively.

**Table 1:** Patient characteristics

Characteristics	Elemental formula group	Control group
Number of subjects, *n*	382	566
Age (years)	62 [45, 74]	63 [45, 75]
Sex, female, *n* (%)	134 (35.1)	204 (36.0)
Charlson comorbidity index	0 [0, 1]	0 [0, 1]
Prognostic factor score	3 [1, 4]	3 [1, 4]
CT severity score	2 [2, 2]	2 [1, 3]
Mechanical ventilation use, *n* (%)	101 (26.4)	201 (35.5)
Renal replacement therapy, *n* (%)	78 (20.4)	125 (22.1)
Vasopressors use, *n* (%)	61 (16.0)	114 (20.1)
Transfusion, *n* (%)	101 (26.4)	158 (27.9)
Annual number of acute pancreatitis per hospital	61.8 [47.7, 85.5]	73.3 [48.2, 92.5]

Numeric variables are expressed as median [25th–75th percentiles]

*CT* computed tomography

### Case-mix adjustment

**Fig. 2:**
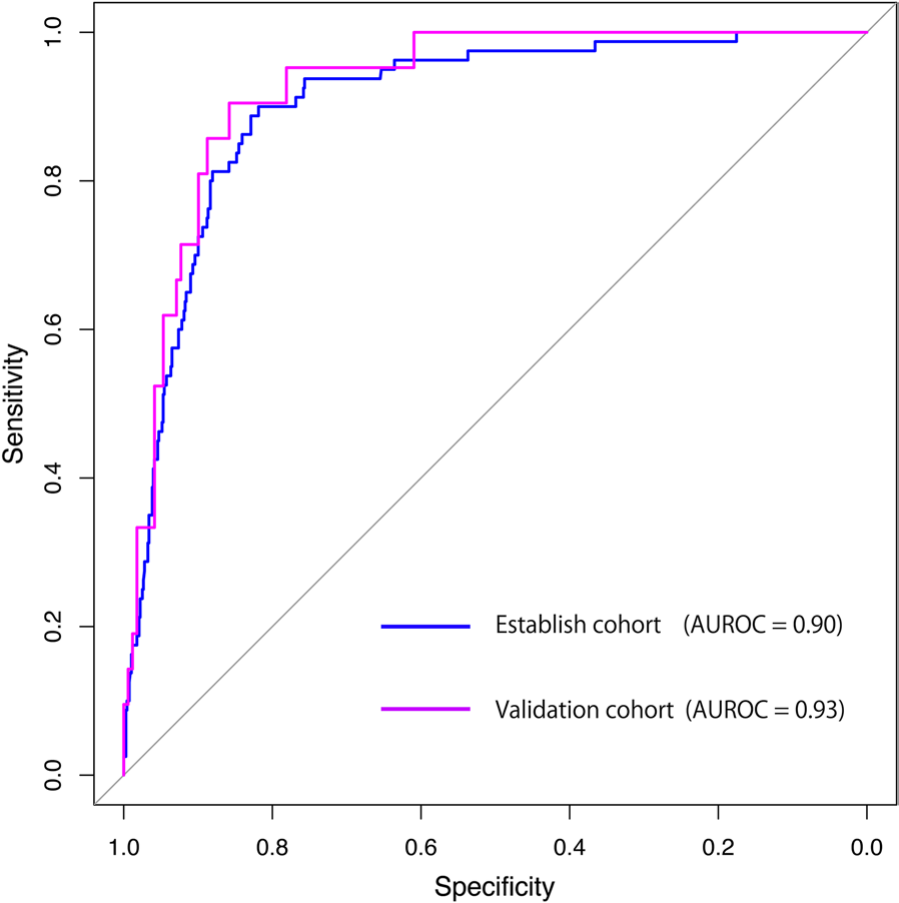
Receiver operating curves of the risk adjustment model in the establishment and validation cohort. *AUROC* area under the receiver operating curve

All VIFs of the variables used in the regression analysis were < 2, which eliminated the issue of multicollinearity in our model. The case-mix classification model that we established had high accuracy regarding in-hospital mortality, with an AUROC of 0.90 for the establishment cohort (Fig. 2). Furthermore, the established model was well calibrated for the validation cohort (AUROC, 0.93; Hosmer–Lemeshow goodness-of-fit test, *p* = 0.704) (Additional file 4).

### Multivariate mixed-effect regression model

Results of the multivariate mixed-effects model are summarized in Table 2. No significant difference was observed for in-hospital mortality [adjusted odds ratio (95% confidence interval; CI) = 0.94 (0.53–1.67)] and all secondary outcomes.

**Table 2:** Results of multivariate mixed-effects regression analysis

Outcomes	Elemental formula (*N* = 382)	Control (*N* = 566)	Adjusted odds ratio [95% CI]	Adjusted difference [95% CI]	*p* value
*Primary outcome*					
In-hospital mortality, %	10.2	11.0	0.94 (0.53–1.67)	–	0.823
*Secondary outcomes*					
Sepsis development, %	5.0	7.1	0.66 (0.34–1.28)	–	0.218
Mean hospital-free days at 90 days, days	54	51	–	2 days (−2 to 5)	0.331
Mean total health-care costs, $	$29,360	$34,214	–	−$4250 (−8643 to 141)	0.872

*CI* confidence interval

### Propensity score matching

Among all 948 eligible patients, 380 propensity score-matched pairs were generated via the matching process. The ASMD in the variables indicated a well-matched balance (Additional file 5). The in-hospital mortality rate was 10.3% (39/380) for the elemental formula group and 8.7% (33/380) for the control group in the propensity score-matched cohort. Results of the propensity score matching analysis are summarized in Table 3. Similar to the results of the multivariate mixed-effects model, no significant difference was observed for in-hospital mortality [adjusted odds ratio (95% confidence interval: CI) = 1.20 (0.74–1.96)] and in all the secondary outcomes.

**Table 3:** Results of propensity score matching analysis

Outcomes	Elemental formula (*N* = 380)	Control (*N* = 380)	Adjusted odds ratio [95% CI]	Adjusted difference [95% CI]	*p* value
*Primary outcome*					
In-hospital mortality, %	10.3	8.7	1.20 (0.74–1.96)	–	0.458
*Secondary outcomes*					
Sepsis development, %	5.0	6.8	0.72 (0.39–1.32)	–	0.284
Mean hospital-free days at 90 days, days	54	53	–	1.1 days (−3.0 to 5.2)	0.596
Mean total health-care costs, $	$29,450	$32,366	–	−$2916 (−8267 to 2435)	0.286

*CI* confidence interval

### Analyses in patients with SAP

The results of analyses using the mixed-effect and propensity score matching models in patients with SAP are presented in Additional file 6. No significant difference was observed for all outcomes in both models as well as in patients with SAP.

## Discussion

In this retrospective cohort study using a Japanese national database, we assessed the efficacy of elemental formula compared to other formulae of enteral nutrition for the outcomes of in-hospital mortality, development of sepsis, hospital-free days, and total health-care costs per admission. The results demonstrated that elemental formula use had few associations with all outcomes regarding early enteral nutrition in the treatment of acute pancreatitis. To the best of our knowledge, this study is the first to use large-scale data to directly compare the impact of elemental formula and other formulae in patients with acute pancreatitis.

Several RCTs demonstrated the superiority of enteral nutrition compared to parenteral nutrition in late 1990s [8–11], resulting in a paradigm shift in the nutritional management of patients with acute pancreatitis toward enteral nutrition. The main mechanism of acute pancreatitis is autodigestion of the pancreas due to self-producing proteases. Enteral feeding increases pancreatic secretion by stimulating the cephalic and gastric phases, and early oral feeding may lead to recurrence of symptoms, elevation of serum amylase and lipase, and delayed complications [23, 24]. On the other hand, it is widely recognized that enteral nutrition decreases gut permeability, reduces bacterial translocation, and activates mucosal immunity [25–27]. Considering the results of clinical studies that suggested the superiority of enteral feeding compared to parenteral feeding for patients with acute pancreatitis, it is expected that the benefit of maintaining gut immunity would overcome the drawbacks of an increase in the exocrine secretions of the pancreas.

Several clinical studies concerning the type of enteral nutrition have been conducted since the 2000s [28–30]; however, there has not been sufficient evidence to justify the use of specific nutrition, such as probiotics, fiber-enriched formulae, and *n* − 3 fatty acids. In response to those results, recent guidelines [31–35] generally recommended the use of standard polymeric formulae; however, the issue of a shortage of sufficient evidence was also mentioned in their comments (Additional file 7). Despite the recommendation of guidelines, in this study, the proportion of patients who received elemental formula reached approximately 40% among patients who received enteral feeding via nasogastric tube. This suggested that clinicians still expect the comparative effectiveness of elemental formula and that further evidence regarding this is required.

A study on volunteers indicated that elemental formula has a less stimulatory effect on the secretion of pancreatic lipase and chymotrypsin compared to food homogenate [36]. Elemental formula is expected to achieve both maintenance of gut immunity and suppression of exocrine secretions in the pancreas, suggesting that elemental formula is theoretically beneficial for patients with acute pancreatitis. However, contrary to this assumption, the results of this study failed to show the superiority of elemental formula compared to semi-elemental and polymeric formulae, and several reasons for this can be considered. It has been reported that trans-jejunal feeding of polymeric formulae was well tolerated by patients with acute pancreatitis and can potentially be used to facilitate pancreatic rest [37]. This means that standard formulae also could achieve pancreatic rest by controlling the route of administration; however, information on the dosage regimen and administration route could not be evaluated in this study. In addition, a study comparing the absorption of nutrients in patients with cystic fibrosis and pancreatic insufficiency who were administered elemental or polymeric formulae with and without pancreatic enzymes showed that no benefit was experienced by patients who were administered elemental formula, compared to those administered the polymeric formula with pancreatic enzymes [38]. This result suggested that patients with pancreatic insufficiency could utilize polymeric formulae as opposed to elemental formulae, with pancreatic enzyme supplementation as needed. Furthermore, a recent experimental study in mice [39] showed the advantage of a semi-elemental formula compared to elemental formula from the perspective of absorption, tolerability, and maintenance of gut immunity. Thus, elemental formula may not always be beneficial compared to a polymeric formula in experimental settings.

Because this study was a retrospective study conducted for a limited duration, the possibility of under power was a concern. Therefore, we performed a sample size calculation for in-hospital mortality based on the effect size estimated in propensity score matching analysis. The results of power analysis, assuming *α* = 0.05 (two-sided) and *β* = 0.2 (power = 80%), showed that 5270 patients in each group were required to demonstrate statistical significance. This result implied that the effect of elemental formula was clinically limited for the outcome of mortality in patients with acute pancreatitis.

The strength of our study was that we directly evaluated the effect of elemental formula in a large number of patients with acute pancreatitis compared to those in previous studies. Furthermore, we used a statistical model with well-calibrated case-mix adjustment, which simultaneously accounted for hospital-level clustering. In addition, health-care costs are uniform across all hospitals and individuals in Japan, which is often not the case in the other countries. However, our study had several limitations. First, the possibility of residual confounding existed because of the retrospective nature of our study; however, the results were sufficiently informative because the case-mix classification model used demonstrated high accuracy for predicting in-hospital mortality. Second, details of the administration route were not recorded in the DPC database; therefore, we could not distinguish between nasogastric feeding and nasojejunal feeding in this study. However, meta-analysis by Chang et al. [40] showed that this difference had no effect on the outcomes. Third, because this was not a parallel RCT, standardization of the nutrition regimen between the study groups was not possible. Therefore, a nutrition management, such as administration dose, rate, and methods (i.e., continuous or intermitted), for each patient varied according to respective patient status and hospital standard operation procedure. Fourth, a number of patients were excluded through a patient selection process, and this may have caused a selection bias. In Japan, proportion of severe acute pancreatitis patients who received enteral feeding within 48 h of admission was reported to be approximately 10% [41]. In addition, because the present study analyzed only patients who received enteral nutrition via nasogastric tube, less severe patients who were allowed oral intake were not analyzed. Furthermore, registering information on the severity of pancreatitis was not mandatory in the DPC database. Finally, although every use of elemental formula product could be specified because they required prescriptions, some products of semi-elemental and polymeric formulae could not be specified in the DPC database because they were treated as meals and did not require prescriptions in Japan. This prevented evaluation of the detailed differences among the type of semi-elemental and polymeric formulae. However, apparent overfeeding was not observed, at least in the elemental formula group, and the duration of enteral feeding was not markedly different among groups.

Despite these limitations, the design of our study that compared elemental formula and other formulae was reasonable to show the difference of the effect of enteral nutrition formulae from the perspective of pancreatic rest, since only elemental formula does not require digestion process. Elemental formula is generally more expensive compared to polymeric formulae; however, the results of this study did not demonstrate any significant difference in outcomes between the two. From the results of this study and previous studies, there is not sufficient evidence to justify the routine use of elemental formula in the initiation of enteral nutrition for patients with acute pancreatitis.

## Conclusions

The results of a retrospective cohort study using a large-scale national database did not show the benefits of elemental formula compared to semi-elemental and polymeric formulae in patients with acute pancreatitis. Further assessment of alternative nutritional strategy is expected.

## Additional files

**Additional file 1.** Severity Scoring System for Acute Pancreatitis of the Japanese Ministry of Health, Labour and Welfare (2008).

**Additional file 2.** International Classification of Diseases, 10th Revision codes used in the study.

**Additional file 3.** Mean prescription dose of elemental formula from the day of admission to day 14.

**Additional file 4.** Calibration plot.

**Additional file 5.** Patient characteristics before and after propensity score matching.

**Additional file 6.** Results of analyses using mixed-effect model and propensity score matching model in patients with severe acute pancreatitis.

**Additional file 7.** Recommendations in clinical practice guidelines for the type of formulae in acute pancreatitis.

## Abbreviations

RCT: randomized controlled trial
DPC: Diagnosis Procedure Combination
ICD-10: International Classification of Diseases, 10th revision
CT: computed tomography
VIF: variance inflation factor
AUROC: area under the receiver operating curve
ASMD: absolute standardized mean difference
SD: standard deviation
SAP: severe acute pancreatitis
CI: confidence interval
