# Supplementary material for: Comparative effectiveness of elemental formula in the early enteral nutrition management of acute pancreatitis: a retrospective cohort study
Source: Ann Intensive Care. 2018 Jun 5;8:69. doi: 10.1186/s13613-018-0414-6 (PMC5986693; doi:10.1186/s13613-018-0414-6)
Supplement: Supplementary file 1 — Additional file 1. Severity Scoring System for Acute Pancreatitis of the Japanese Ministry of Health, Labour and Welfare (2008). [file 13613_2018_414_MOESM1_ESM.doc]

| **Additional file 1. Severity Scoring System for Acute Pancreatitis of the Japanese Ministry of Health, Labour and Welfare (2008)** | | |
| --- | --- | --- |
| **Prognostic factors (1 point for each factor)** | | |
| 1. Base excess ≤3 mEq/L or shock (systolic blood pressure <80 mmHg) | | |
| 2. PaO_2_ ≤60 mmHg (room air) or respiratory failure (respirator management is needed) | | |
| 3. BUN ≥40 mg/dL (or creatinine ≥2.0 mg/dL) or oliguria (daily urine output <400 mL even after intravenous fluid resuscitation) | | |
| 4. LDH ≥2 times of upper limit of normal | | |
| 5. Platelet count ≤100,000/mm^3^ | | |
| 6. Serum Ca ≤7.5 mg/dL | | |
| 7. CRP ≥15 mg/dL | | |
| 8. Number of positive measures in SIRS criteria ≥3 | | |
| 9. Age ≥70 years | | |
| **CT grade by contrast-enhanced CT** | | |
| 1. Extrapancreatic progression of inflammation | | |
|  | Anterior pararenal space | **0 point** |
|  | Root of mesocolon | **1 point** |
|  | Beyond lower pole of kidney | **2 points** |
| 2. Hypoenhanced lesion of the pancreas [The pancreas is conveniently divided into three segments (head, body, and tail)] | | |
|  | Localized in each segment or only surrounding the pancreas | **0 point** |
|  | Covers 2 segments | **1 point** |
|  | Occupies entire 2 segments or more | **2 points** |
| 1 + 2 = Total scores | | |
|  | Total score = 0 point or 1 point | **Grade 1** |
|  | Total score = 2 points | **Grade 2** |
|  | Total score = 3 points | **Grade 3** |
| **Assessment of severity** | | |
| If prognostic factors are scored as 3 points or more, or (2) If CT grade is judged as 2 or more, the patient is evaluated to be ‘‘severe acute pancreatitis’’. | | |
| Measures in SIRS diagnostic criteria: (1) temperature, >38°C or <36°C; (2) heart rate, >90 beats/min; (3) ventilatory rate, >20 breaths/min or PaCO2 <32 torr; (4) White blood cell, >12,000 cells/mm^3^, <4000 cells/mm^3^, or >10% immature (band) forms.  PaO_2_, partial pressure of oxygen in blood; BUN, blood urea nitrogen; LDH, lactate dehydrogenase; CRP, C-reactive protein; SIRS, systemic inflammatory response syndrome. | | |
