# Supplementary material for: Comparative effectiveness of elemental formula in the early enteral nutrition management of acute pancreatitis: a retrospective cohort study
Source: Ann Intensive Care. 2018 Jun 5;8:69. doi: 10.1186/s13613-018-0414-6 (PMC5986693; doi:10.1186/s13613-018-0414-6)
Supplement: Supplementary file 2 — Additional file 2. International Classification of Diseases, 10th Revision codes used in the study. [file 13613_2018_414_MOESM2_ESM.doc]

| **Additional file 2**. **International Classification of Diseases, 10th Revision codes used in the study.** | |
| --- | --- |
| Disease | ICD-10 codes |
| Acute pancreatitis | K85 |
| Sepsis | A201, A207, A227, A267, A327, A40, A400, A401, A402, A403, A408, A409, A41, A410, A411, A412, A413, A414, A415, A418, A419, A427, B377 |
| ICD-10, International Classification of Diseases, 10th Revision. | |
