# Supplementary material for: Comparative effectiveness of elemental formula in the early enteral nutrition management of acute pancreatitis: a retrospective cohort study
Source: Ann Intensive Care. 2018 Jun 5;8:69. doi: 10.1186/s13613-018-0414-6 (PMC5986693; doi:10.1186/s13613-018-0414-6)
Supplement: Supplementary file 3 — Additional file 3. Mean prescription dose of elemental formula from the day of admission to day 14. [file 13613_2018_414_MOESM3_ESM.doc]

**Additional file 3. Mean prescription dose of elemental formula from the day of admission to day 14.**


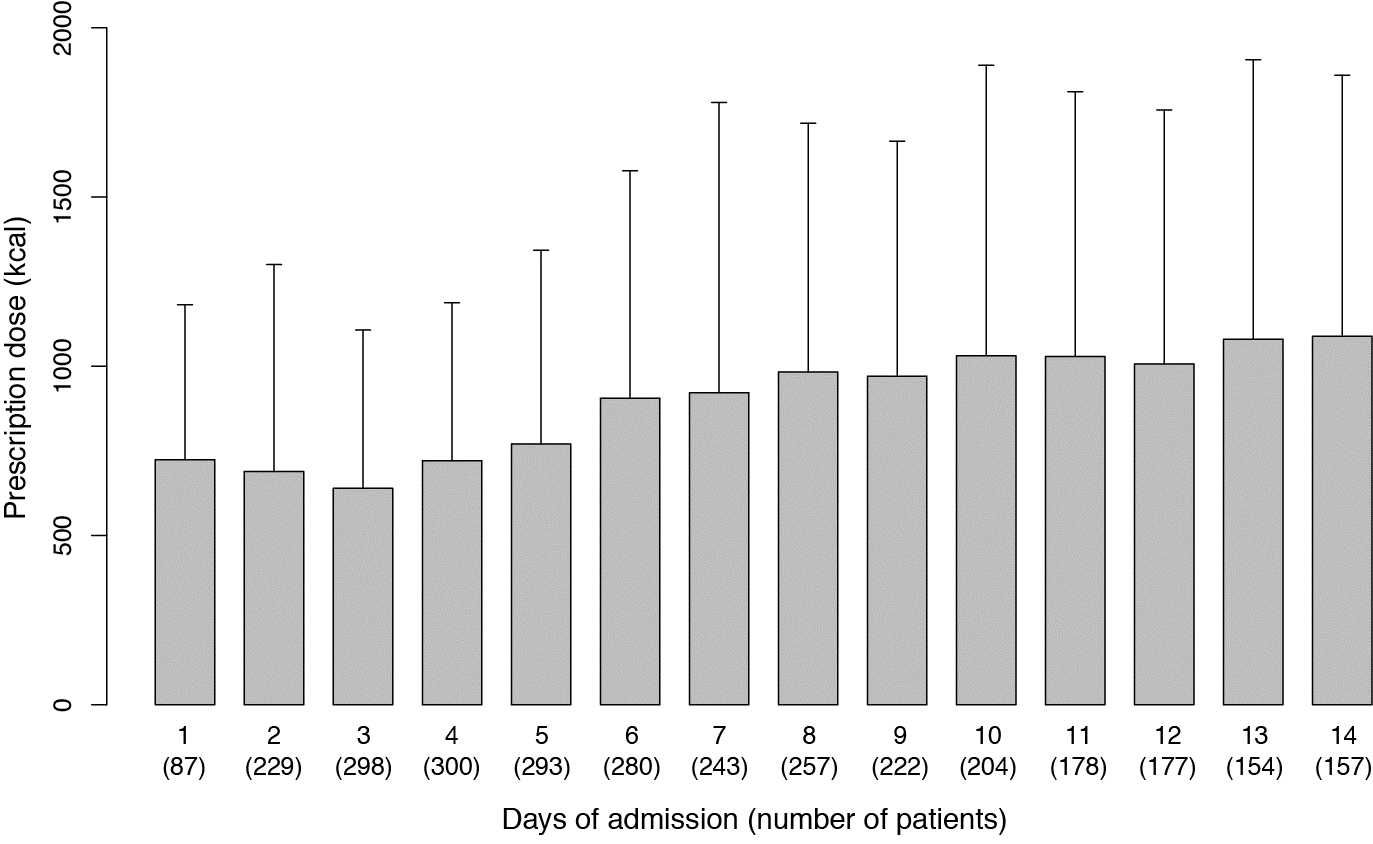


Error bar indicates standard deviation. Number in parentheses shows the number of patients who were prescribed elemental formula.
