# Supplementary figures and images for: Comparative effectiveness of elemental formula in the early enteral nutrition management of acute pancreatitis: a retrospective cohort study
Source: Ann Intensive Care. 2018 Jun 5;8:69. doi: 10.1186/s13613-018-0414-6 (PMC5986693; doi:10.1186/s13613-018-0414-6)

**Additional file 4. Calibration plot**


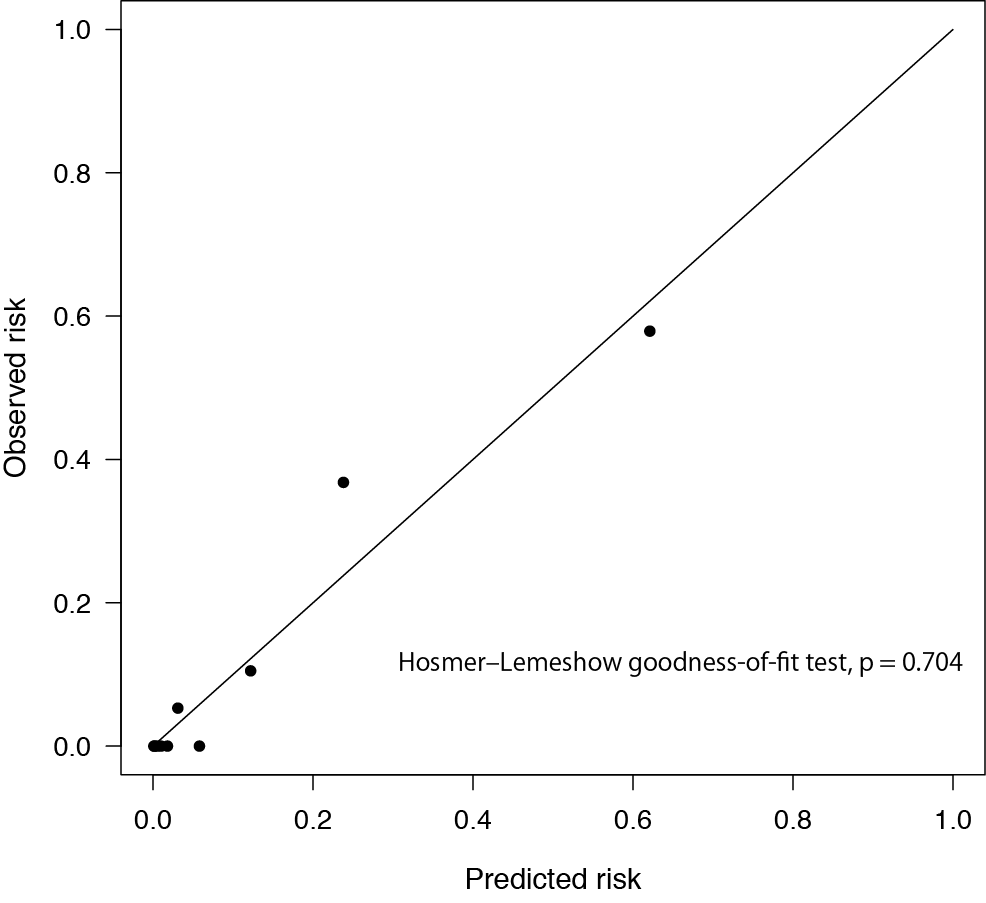

Supplement: Supplementary file 4 — Additional file 4. Calibration plot. [file 13613_2018_414_MOESM4_ESM.doc]
