# Supplementary material for: Comparative effectiveness of elemental formula in the early enteral nutrition management of acute pancreatitis: a retrospective cohort study
Source: Ann Intensive Care. 2018 Jun 5;8:69. doi: 10.1186/s13613-018-0414-6 (PMC5986693; doi:10.1186/s13613-018-0414-6)
Supplement: Supplementary file 5 — Additional file 5. Patient characteristics before and after propensity score matching. [file 13613_2018_414_MOESM5_ESM.doc]

| **Additional file 5. Patient characteristics before and after propensity score matching** | | | | | | |
| --- | --- | --- | --- | --- | --- | --- |
|  | Unmatched cohort | | | Matched cohort | | |
| Variables | Elemental diet | Control | ASMD | Elemental diet | Control | ASMD |
| Number of subjects, n | 382 | 566 | – | 380 | 380 | – |
| Age (years) | 62 [45, 74] | 63 [45, 75] | 0.07 | 62 [45, 74] | 61 [44, 74] | 0.01 |
| Sex, female, n (%) | 134 (35.1) | 204 (36.0) | 0.02 | 134 (35.3) | 129 (34.9) | 0.03 |
| Charlson comorbidity index | 0 [0, 1] | 0 [0, 1] | 0.05 | 0 [0, 1] | 1 [0, 1] | 0.01 |
| Prognostic factor score | 3 [1, 4] | 3 [1, 4] | 0.07 | 3 [1, 4] | 3 [1, 4] | 0.00 |
| CT severity score | 2 [2, 2] | 2 [1, 3] | 0.08 | 2 [2, 2] | 2 [2, 3] | 0.01 |
| Mechanical ventilation use, n (%) | 101 (26.4) | 201 (35.5) | 0.20 | 101 (26.6) | 102 (33.8) | 0.01 |
| Renal replacement therapy, n (%) | 78 (20.4) | 125 (22.1) | 0.07 | 78 (20.5) | 83 (36.5) | 0.03 |
| Vasopressors use, n (%) | 61 (16.0) | 114 (20.1) | 0.04 | 61 (16.1) | 60 (22.5) | 0.01 |
| Transfusion, n (%) | 101 (26.4) | 158 (27.9) | 0.03 | 100 (26.3) | 100 (35.3) | 0.00 |
| Annual number of cases of acute pancreatitis per hospital | 61.8 [47.7, 85.5] | 73.3 [48.2, 92.5] | 0.20 | 61.8 [47.7, 85.5] | 65.5 [47.4, 86.5] | 0.01 |
| Numeric variables are expressed as median [25^th^–75^th^ percentiles]  ASMD, Absolute Standardised mean difference; CT, computed tomography | | | | | | |
