# Supplementary material for: Comparative effectiveness of elemental formula in the early enteral nutrition management of acute pancreatitis: a retrospective cohort study
Source: Ann Intensive Care. 2018 Jun 5;8:69. doi: 10.1186/s13613-018-0414-6 (PMC5986693; doi:10.1186/s13613-018-0414-6)
Supplement: Supplementary file 6 — Additional file 6. Results of analyses using mixed-effect model and propensity score matching model in patients with severe acute pancreatitis. [file 13613_2018_414_MOESM6_ESM.doc]

**Additional file 6. Results of analyses using mixed-effect model and propensity score matching model in patients with severe acute pancreatitis**

*Study population*

During the study period, 817 patients with severe acute pancreatitis were identified. Of these, 339 patients were administered an elemental formula. Characteristics of patients with severe acute pancreatitis according to the type of enteral nutritional formulae administered was presented below.

| **Characteristics of patients with severe acute pancreatitis** | | |
| --- | --- | --- |
| Characteristics | Elemental formula group | Control group |
| Number of subjects, n | 339 | 478 |
| Age (years) | 61 [45, 73] | 62 [44, 74] |
| Sex, Female, n (%) | 121 (35.7) | 167 (36.0) |
| Charlson comorbidity index | 0 [0, 1] | 0 [0, 1] |
| Prognostic factor score | 3 [2, 4] | 3 [2, 5] |
| CT severity score | 2 [2, 3] | 2 [2, 3] |
| Mechanical ventilation use, n (%) | 97 (28.6) | 181 (35.5) |
| Renal replacement therapy, n (%) | 75 (22.1) | 117 (22.1) |
| Vasopressors use, n (%) | 59 (17.4) | 104 (20.1) |
| Transfusion, n (%) | 95 (28.0) | 140 (27.9) |
| Annual number of acute pancreatitis per hospital | 63.6 [47.7, 85.5] | 76.5 [49.7, 92.5] |
| Numeric variables are expressed as median [25^th^–75^th^ percentiles]  CT, computed tomography | | |

The in-hospital mortality rate was 10.9% (37/339) in the elemental formula group and 12.1% (58/478) in the control group.

*Multivariate mixed-effect regression model*

Results of the multivariate mixed-effects model are summarized in below. No significant difference was observed for in-hospital mortality [adjusted odds ratio (95% confidence interval; CI) = 0.94 (0.53–1.67)] and in all the secondary outcomes.

| **Results of multivariate mixed-effects regression analysis in patients with severe acute pancreatitis** | | | | | | | | |
| --- | --- | --- | --- | --- | --- | --- | --- | --- |
| Outcomes | | Elemental formula  (N = 339) | Control  (N = 478) | | Adjusted odds ratio  [95% CI] | | Adjusted difference  [95% CI] | *p* value |
| Primary outcome | | | | | | | | |
|  | In-hospital mortality, % | 10.9 | | 12.1 | | 0.92 (0.50–1.67) | – | 0.775 |
| Secondary outcomes | | | | | | | | |
|  | Sepsis development, % | 5.0 | | 8.2 | | 0.55 (0.28–1.09) | – | 0.089 |
|  | Mean hospital-free days at 90 days, days | 53 | | 49 | | – | 2 (˗2–5) | 0.368 |
|  | Mean total healthcare costs, $ | 30,881 | | 36,560 | | – | ˗4566 (˗9503–371) | 0.935 |
| CI, confidence interval | | | | | | | | |

*Propensity score matching*

Among all 817 eligible patients, 338 matched pairs were generated via the matching process. Patients characteristics before and after propensity score matching are presented in below. The absolute standardized mean difference in the variables indicated a well-matched balance.

| **Characteristics in patients with severe acute pancreatitis before and after propensity score matching** | | | | | | |
| --- | --- | --- | --- | --- | --- | --- |
|  | Unmatched cohort | | | Matched cohort | | |
| Variables | Elemental formula | Control | ASMD | Elemental formula | Control | ASMD |
| Number of subjects, n | 339 | 478 | – | 338 | 338 | – |
| Age (years) | 61 [45, 73] | 62 [44, 74] | 0.05 | 61 [45, 73] | 62 [44.3, 73.8] | 0.03 |
| Sex, Female, n (%) | 121 (35.7) | 167 (36.0) | 0.02 | 120 (35.5) | 113 (33.4) | 0.04 |
| Charlson comorbidity index | 0 [0, 1] | 0 [0, 1] | 0.04 | 0 [0, 1] | 1 [0, 1] | 0.00 |
| Prognostic factor score | 3 [2, 4] | 3 [2, 5] | 0.13 | 3 [2, 4] | 3 [2, 4] | 0.02 |
| CT severity score | 2 [2, 3] | 2 [2, 3] | 0.00 | 2 [2, 3] | 2 [2, 3] | 0.03 |
| Mechanical ventilation use, n (%) | 97 (28.6) | 181 (35.5) | 0.20 | 97 (28.7) | 97 (28.7) | 0.00 |
| Renal replacement therapy, n (%) | 75 (22.1) | 117 (22.1) | 0.06 | 74 (21.9) | 66 (19.5) | 0.06 |
| Vasopressors use, n (%) | 59 (17.4) | 104 (20.1) | 0.11 | 59 (17.5) | 60 (17.8) | 0.01 |
| Transfusion, n (%) | 95 (28.0) | 140 (27.9) | 0.03 | 94 (27.8) | 92 (27.2) | 0.01 |
| Annual number of acute pancreatitis per hospital | 63.6 [47.7, 85.5] | 76.5 [49.7, 92.5] | 0.19 | 63.6- [47.8, 85.5] | 65.5 [47.4, 86.5] | 0.03 |
| Numeric variables are expressed as median [25^th^–75^th^ percentiles]  ASMD, Absolute standardized mean difference; CT. computed tomography | | | | | | |

Regarding the propensity score-matched cohort, in-hospital mortality rate was 10.9% (37/338) for the elemental diet group and 10.1% (34/338) for the control group. Results of the propensity score matching analysis are summarized below. Similar to the results of the multivariate mixed-effects model, no significant difference was observed for in-hospital mortality [adjusted odds ratio (95% CI) = 1.10 (0.67–1.80)] and in all of the secondary outcomes.

| **Results of propensity score matching analysis in patients with severe acute pancreatitis** | | | | | | | | |
| --- | --- | --- | --- | --- | --- | --- | --- | --- |
| Outcomes | | Elemental formula  (N = 338) | Control  (N = 338) | | Adjusted odds ratio  [95% CI] | | Adjusted difference  [95% CI] | *p*-value |
| Primary outcome | | | | | | | | |
|  | In-hospital mortality, % | 10.9 | | 10.1 | | 1.10 (0.67 to 1.80) | – | 0.707 |
| Secondary outcomes | | | | | | | | |
|  | Sepsis development, % | 5.0 | | 7.4 | | 0.66 (0.35 to 1.25) | – | 0.205 |
|  | Mean hospital-free days at 90 days, days | 53 | | 52 | | – | 0 (–4 to 5) | 0.846 |
|  | Mean total healthcare costs, $ | 30,914 | | 33,652 | | – | –2738 (–8403 to 2927) | 0.344 |
| Abbreviation: CI, confidence interval. | | | | | | | | |
