# Supplementary material for: Comparative effectiveness of elemental formula in the early enteral nutrition management of acute pancreatitis: a retrospective cohort study
Source: Ann Intensive Care. 2018 Jun 5;8:69. doi: 10.1186/s13613-018-0414-6 (PMC5986693; doi:10.1186/s13613-018-0414-6)
Supplement: Supplementary file 7 — Additional file 7. Recommendations in clinical practice guidelines for the type of formulae in acute pancreatitis. [file 13613_2018_414_MOESM7_ESM.doc]

| **Additional file 7. Recommendations in clinical practice guidelines for the type of formulae in acute pancreatitis.** | | |
| --- | --- | --- |
| Guidelines | Recommendation | Comment |
| ESPEN (2006) [31] | Peptide-based formulae can be used safely (Grade A) | Most trials (human and animal) have been carried out using peptide-based formula, which can therefore be recommended for feeding. |
| American College of Gastroenterology (2013) [32] | In mild AP, initiation of feeding with a low-fat solid diet appears as safe as a clear liquid diet (conditional recommendations, moderate quality of evidence). | Oral feedings introduced in mild AP do not need to begin with clear liquids and increase in a stepwise manner, but may begin as a low-residue, low-fat, so diet when the patient appears to be improving. |
| Japanese guidelines for the management of acute pancreatitis (2015) [33] | Enteral nutrition can be provided from among digestible nutrients, semi‐digestible nutrients and component nutrients, considering the viscosity and osmotic pressure. (Grade B) | While type of diet was not specifically examined in the technical review, success of early feeding has been demonstrated using a variety of diets including low-fat, normal fat, and soft or solid consistency, and thus starting with a clear liquid diet is not required. |
| SCCM/ASPEN (2016) [34] | We suggest using a standard polymeric formula to initiate enteral nutrition in the patient with severe acute pancreatitis. Although promising, the data are currently insufficient to recommend placing a patient with severe acute pancreatitis on an immune-enhancing formulation at this time. (Quality of Evidence: Very Low) | A standard polymeric formula is appropriate for initiating early enteral nutrition for patients with moderate to severe acute pancreatitis. |
| AGA (2018) [35] | No statement | While type of diet was not specifically examined in the technical review, success of early feeding has been demonstrated using a variety of diets including low-fat, normal fat, and soft or solid consistency,21 and thus starting with a clear liquid diet is not required. |
| Abbreviations: AP, acute pancreatitis; ESPEN, European Society for Clinical Nutrition and Metabolism; SCCM, Society of Critical Care Medicine; ASPEN, American Society for Parenteral and Enteral Nutrition; AGA, American Gastroenterological Association | | |
